# A study of PD-L1 expression in *KRAS* mutant non-small cell lung cancer cell lines exposed to relevant targeted treatments

**DOI:** 10.1371/journal.pone.0186106

**Published:** 2017-10-05

**Authors:** Anna Minchom, Parames Thavasu, Zai Ahmad, Adam Stewart, Alexandros Georgiou, Mary E. R. O’Brien, Sanjay Popat, Jaishree Bhosle, Timothy A. Yap, Johann de Bono, Udai Banerji

**Affiliations:** 1 The Lung Unit, Department of Medicine, The Royal Marsden, Sutton, United Kingdom; 2 The Drug Development Unit, The Institute of Cancer Research and The Royal Marsden, London, United Kingdom; 3 The Lung Unit, The Royal Marsden, London, United Kingdom; Universita degli Studi di Parma, ITALY

## Abstract

We investigated PD-L1 changes in response to MEK and AKT inhibitors in *KRAS* mutant lung adenocarcinoma (adeno-NSCLC). PD-L1 expression was quantified using immunofluorescence and co-culture with a jurkat cell-line transfected with NFAT-luciferase was used to study if changes in PD-L1 expression in cancer cell lines were functionally relevant. Five *KRAS* mutant cell lines with high PD-L1 expression (H441, H2291, H23, H2030 and A549) were exposed to GI_50_ inhibitor concentrations of a MEK inhibitor (trametinib) and an AKT inhibitor (AZD5363) for 3 weeks. Only 3/5 (H23, H2030 and A549) and 2/5 cell lines (H441 and H23) showed functionally significant increases in PD-L1 expression when exposed to trametinib or AZD5363 respectively. PD-L1 overexpression is not consistent and is unlikely to be an early mechanism of resistance to *KRAS* mutant adeno-NSCLC treated with MEK or AKT inhibitors.

## Introduction

Lung cancer is the leading cause of cancer death in developed countries with 1.8 million new lung cancer diagnoses occurring globally per annum [1]. Standard of care options for metastatic NSCLC have evolved from chemotherapy doublets to targeted treatments such as EGFR and ALK inhibitors in defined subsets of patients [2] [3] [4] [5] and, most recently, immune checkpoint modulating drugs [6] [7].

Programmed-death 1 and 2 (PD-1 and PD-2) are cell-surface receptors found on T-cells and their interaction with programmed-death ligand 1 and 2 (PD-L1 and PD-L2) on cancer cells provide an important inhibitory role in the immune response [8]. PD-1 inhibitors have shown benefit in the first and second-line setting in both squamous and adenocarcinoma of the lung (adeno-NSCLC) [7] [9] [10].

*KRAS* mutations are found in 33% of advanced adeno-NSCLC [11]. There are currently no drugs in clinical evaluation that directly inhibit KRAS. Attempts to use MEK inhibition or PI3K pathway inhibition alone have failed [12] [13]. Despite promising activity in combination with chemotherapy, in randomized phase 2 studies the efficacy of MEK inhibitors in combination with chemotherapy has not been proven [14] [15]. Drug combinations targeting dual aspects of the MEK and PI3K pathways are under evaluation and show promising results [16].

Studies have demonstrated PD-L1 up regulation with *KRAS* mutations [17] [18]. The pathway responsible for this is not fully elucidated. Lastwika et al, in a study of cell lines and mouse models including *KRAS* mutant models, demonstrated PD-L1expression to be via AKT–mTOR signalling [17]. Others have demonstrated up regulation of PD-L1 in the context of *KRAS* mutation through ERK but not AKT [18]. Given PD-L1 expression is up regulated in *KRAS* mutation, with targeted inhibition decreasing PD-L1 expression [17, 18], PD-L1 increase could be proposed as an escape mechanism (and this potentially have a role is secondary resistance) following exposure to targeted agents. This study uses prolonged targeted drug exposure and assessment of PD-L1 expression to assess the potential for PD-L1 in the context of MEK and AKT inhibition in *KRAS* mutant cell lines.

## Materials and methods

### Cell culture and drugs

Cell lines were obtained from ATCC (LGC, Teddington, UK). Cell lines were cultured in RPMI supplemented with 20% fetal bovine serum and incubated in humidified atmosphere with 5% CO_2_. Sensitivity of *KRAS* mutant cell lines to Trametinib (MEK inhibitor) or AZD5363 (AKT inhibitor) (Selleckchem, UK) including determination of GI_50_ was done using 72 hour sulforhodamine B assays. Cells were exposed to GI_50_ concentrations of trametinib and AZD5363 for 6hrs, 24hrs and 3 weeks.

### Immunofluorescence

Fixation was achieved using methanol and 4% formalin followed by antigen retrieval (ventana antigen retrieval solution (Roche, Basel)). A blocking step using bovine serum albumin (BSA), glycine and 0.02% triton for membrane permeabilisation, one hour, was performed. Anti-PD-L1 antibody (rabbit anti-PD-L1 ab58810 [abcam, Cambridge]), anti-rabbit (goat alexa fluor 555 A21429 [Cell Signaling Technology, Danvers, MA]) and anti-pan-cytokeratin (mouse alexa fluor 488, 4523S [Cell Signaling Technology, Danvers, MA]) at 1:200 for 1 hour provided PD-L1 and cytokeratin labelling. Slides were mounted with Molecular Prolong, prolonged diamond antifade with DAPI (Fisher Scientific, Waltham, MA) for nuclear identification. Analysis was using Bioview analysis-linked immunofluorescence microscope system (Bioview, Rehovot, Israel). IN cell software (GE Healthcare Life Sciences, Little Chalfont, UK) quantified fluorescent intensity of control and drugged samples.

### PD-1/NFAT reporter- jurkat cell assay

A jurkat cell line assay system (stably transfected with NFAT-luciferase reporter and human PD-1) was obtained from BPS Biosciences (San Diego, CA). Co-culture of the PD-1/NFAT reporter- jurkat cell line and a PD-L1 expressing cell line results in activation of NFAT-luciferase via TCR and MHC interaction. PD-L1 to PD-1 interaction downregulates the NFAT-luciferase. Thus the degree of decrease in luminescence is proportional to expression of PD-L1.

Cell lines (at a cell count of 35,000) were co-cultured with the jurkat cell line assay system (at a cell count of 20,000) for 16 hours and the one-step luciferase assay system (Biosciences; San Diego, CA) added. Luminescence was measured on a luminescence plate reader (TopCount NXT TM, Packard Parkin Elmer; Meriden, CT). Results were produced in triplicate.

Validation of the co-culture system was performed using a PD-L1 neutralising antibody (#71213; BPS Biosciences, San Diego, CA) (see supplementary material). Western blotting was performed to quantify MHC class I expression on cell lines (see supplementary material).

## Results

### Baseline expression of PD-L1 in *KRAS* mutant cell lines

**Fig 1:**
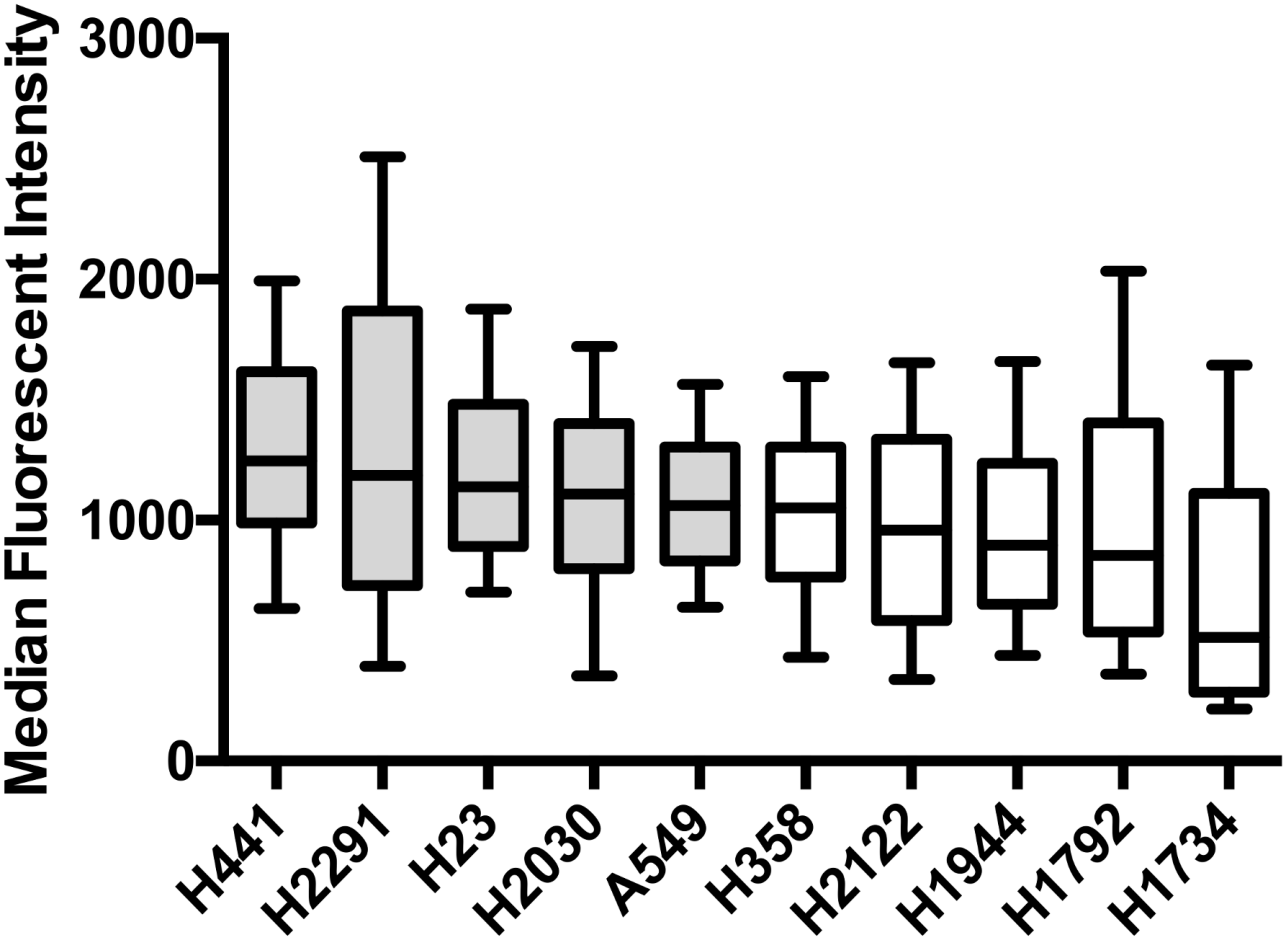
Baseline expression of *KRAS* mutant cell lines. Box and whisker plot. Whiskers representing 10-90^th^ percentile.

PD-L1 expression was quantified in a panel of 10 *KRAS* mutant cell lines (Fig 1). H441, H2291, H2030, H23 and A549 cell lines were chosen for further experiments of AKT and MEK inhibitor exposure as they showed the highest expression of PD-L1 and baseline expression was reproducibly detectable.

### PD-L1 change on exposure to MEK and AKT inhibitors in *KRAS* mutant cell lines

**Fig 2:**
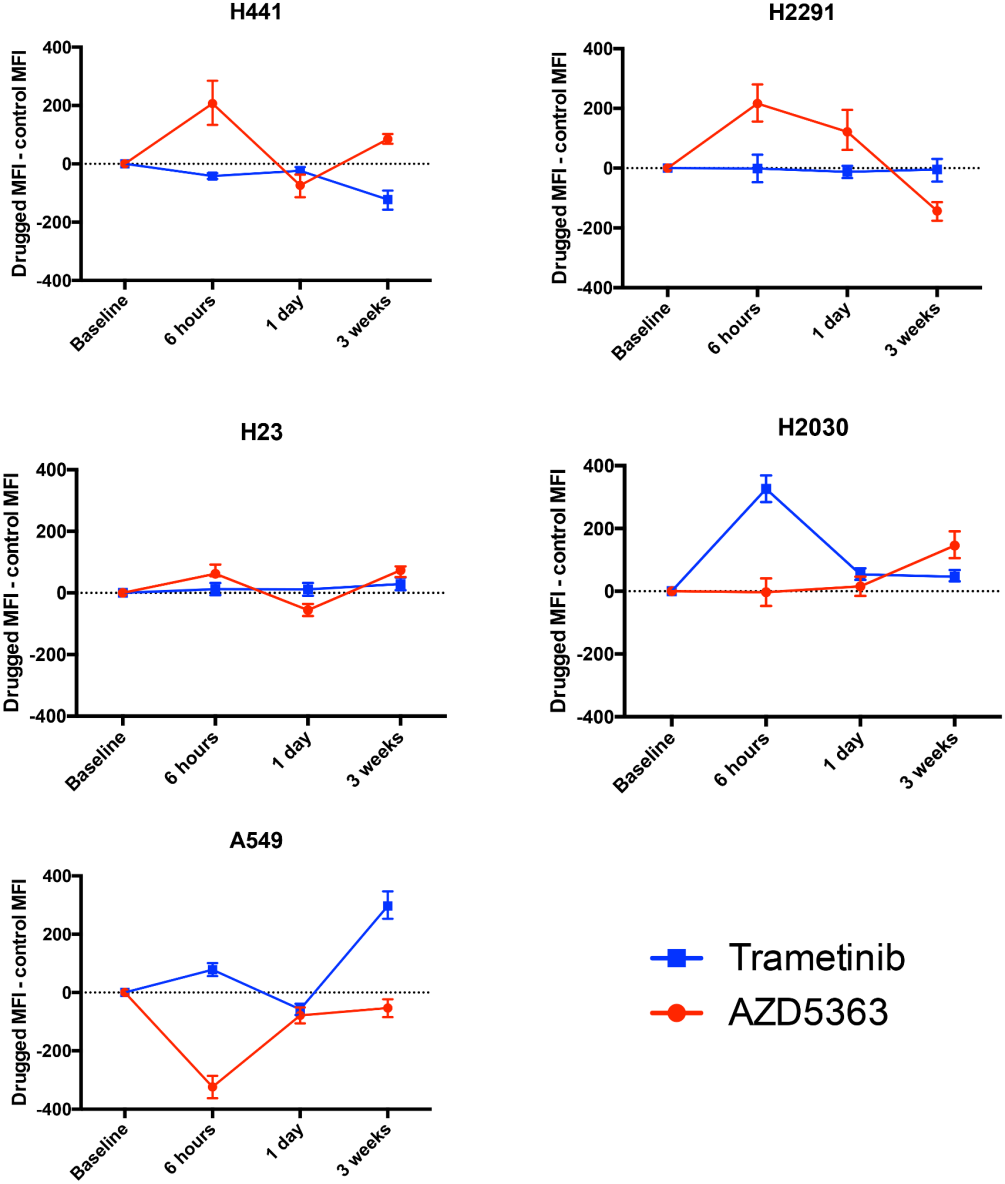
Median fluorescent intensity (MFI) of drugged sample minus the median fluorescent intensity of the control sample for each timepoint with 95% confidence intervals (using Hodges-Lehmann estimated medians for comparison).

The GI_50_ concentrations of the cell lines are documented in supporting information Table 1. The five chosen cell lines were exposed to trametinib or AZD5363 at GI_50_ dose for 3 weeks and the PD-L1 expression quantified by immunofluorescence and expressed in comparison to control (Fig 2 and Table 1). In the cell line panel studied, at 3 weeks when exposed to trametinib, 3/5 cell lines (H23, H2030 and A549) showed a modest but significant increase in PD-L1 expression, 1/5 cells (H441) showed a significant decrease in PD-L1 and 1/5 cell line (H2291) showed no significant change. When exposed to AZD5363, 3/5 cell lines (H441, H23 and H2030) showed a modest but significant increase in PD-L1 and 2/5 (H2291 and A549) showed a small but significant reduction of PD-L1 expression.

**Table 1:** Ratio of median fluorescent intensity of Trametinib and AZD 5363 drugged:control sample and ratio of PD-1/NFAT reporter- jurkat assay luminescence drugged:control sample at each time point with comparison by Mann-Whitney test, p-values<0.05 are highlighted.

	Time point	Immunofluorescence	PD-1/NFAT reporter- jurkat assay
**Trametinib**			
H441	6 hours	0.85 (p<0.0001)	
	1 day	0.93 (p = 0.0003)	
	3 weeks	0.55 (p<0.0001)	1.02 (p = 0.8884)
H2291	6 hours	1.02 (p = 0.9630)	
	1 day	0.96 (p = 0.2205)	
	3 weeks	0.64 (p = 0.8246)	1.22 (p = 0.0142)
H23	6 hours	1.03 (p = 0.2138)	
	1 day	1.01 (p = 0.2702)	
	3 weeks	1.10 (p = 0.0042)	0.93 (p = 0.0188)
H2030	6 hours	1.53 (p<0.0001)	
	1 day	1.28 (p<0.0001)	
	3 weeks	1.23 (p<0.0001)	0.75 (p = 0.0111)
A549	6 hours	1.20 (p<0.0001)	
	1 day	0.92 (p<0.0001)	
	3 weeks	2.42 (p<0.0001)	0.98 (p = 0.6991)
**AZD5363**			
H441	6 hours	1.11 (p<0.0001)	
	1 day	0.94 (p<0.0001)	
	3 weeks	1.38 (p<0.0001)	0.86 (p = 0.0260)
H2291	6 hours	1.73 (p<0.0001)	
	1 day	1.46 (p<0.0001)	
	3 weeks	0.52 (p<0.0001)	0.61 (p = 0.0002)
H23	6 hours	1.11 (p<0.0001)	
	1 day	0.89 (p<0.0001)	
	3 weeks	1.35 (p<0.0001)	0.76 (p = 0.0360)
H2030	6 hours	1.03 (p = 0.9027)	
	1 day	1.07 (p = 0.3133)	
	3 weeks	1.90 (p<0.0001)	0.94 (p = 0.1996)
A549	6 hours	0.73 (p<0.0001)	
	1 day	0.90 (p<0.0001)	
	3 weeks	0.86 (p = 0.0006)	1.12 (p = 0.0012)

### PD-L1 change on PD-1/NFAT reporter- jurkat cell line co-culture on exposure to MEK and AKT inhibitors in *KRAS* mutant cell lines

All cell lines expressed MHC class I, though A549 expressed to a lesser degree than the other cell lines (see supporting information). There were some temporal differences in the expression of PD-L1 at different time points, i.e. 6hrs, 24hrs and 3 weeks. However, we conducted experiments to evaluated functional aspects of PD-L1 expression only at 3 weeks as 3 weeks was considered to be a more relevant time point while exploring mechanisms of resistance to targeted small molecule drugs. All cell lines demonstrate PD-1/NFAT reporter- jurkat cell co-culture results in concordance with the immunofluorescence results with a decrease in luminescence corresponding to an increase in PD-L1 levels on immunofluorescence apart from H2291 on dosing with AZD5363 (Table 1). Thus at 3 weeks when exposed to a MEK and a AKT inhibitor, 3/5 and 2/5 cell lines showed increased expression of PD-L1 which was functionally significant.

## Discussion and conclusions

Previous work has explored mechanisms of interaction between cellular pathways and immune pathways in *EGFR* mutant and *ALK* rearranged NSCLC cancers exposed to EGFR and ALK inhibitors. Azuma and coworkers demonstrated downregulation of PD-L1 by erlotinib when assessed by flow cytometry [19]. A study using western blots, real-time polymerase chain reaction, immunofluorescence, flow cytometry, cell apoptosis assays, cell viability assays, and ELISAs found EGFR activation induced PD-L1 expression through p-ERK1/2/p-c-Jun but not through p-AKT/p-S6 pathway. EGFR activation induced the apoptosis of T cells through the PD-L1/PD-1 axis in tumor cells and peripheral blood mononuclear cells coculture system [20]. Forced expression of *EML4-ALK* in Ba/F3 cells increased PD-L1 expression. PD-L1 expression in *EML4-ALK*-positive NSCLC cells was reduced by treatment with the ALK inhibitor alectinib and by RNA interference with ALK siRNAs. [21]. Data is less clear in the situation of *KRAS* mutations—studies demonstrate PD-L1 up regulation with *KRAS* mutations and potential mechanisms to be via AKT–mTOR signalling or ERK [17] [18].

Signaling changes are known to occur acutely, often in the order of minutes. [22] [23] PD-L1 expression is up regulated in *KRAS* mutation, with targeted inhibition decreasing PD-L1 expression [17] [18]. Previous work has suggested that these changes occur within 2 hours in cell lines [17]. In this experiment we aim to explore the later interaction potentially occurring with the immune pathways that could contribute to resistance to targeted agents rather that signaling changes or acute phase interaction with the immune pathways. Thus the choice of the three week timepoint. More prolonged drug exposures increase the potential for multiple resistance mechanisms including genetic mutations and so were not used.

Our data revealed a small but functional increase and decrease in PD-L1 expression in 3/5 and 1/5 cell lines exposed to the MEK inhibitor trametinib respectively. Further when exposed to the AKT inhibitor AZD5363, functional increase and decrease in PD-L1 expression occurred in 2/5 and 2/5 cell lines respectively. Thus in the *KRAS* mutant cell line panel studied, PD-L1 overexpression is not a consistently seen in response to MEK and AKT inhibitors.

Our approach of in-vitro exposure of cells to MEK and AKT inhibitors with functional read out of T cell activation allowed us to test PDL-1 expression as a putative mechanism of resistance in a significant number of *KRAS* mutant cell lines relatively rapidly and economically. Functional validation of the jurkat co-culture system is provided by the high concordance seen between the immunofluorescence results and jurkat co-culture results. The functional nature of the jurkat co-culture system mitigate the inherent issues with immunofluorescence measurement alone. Immunofluorescence provides only a comparative measure of the uptake of antibodies by surface markers and makes no assessment of the functional interactions of PD-L1, which are the more significant elements when we consider potential biological function and resistance mechanisms. Immunohistochemistry is the most common method of measuring PD-L1 in the clinical context with well documented issues with standardization across platforms [24] and we propose that the jurkat co-culture system be investigated in the clinical context. This could involve the isolation of cancer cells from circulating tumour cell platform or serous effusions [25] [26] and is worthy of further investigation. We recognize the experiments in immunocompetent animal models will offer the most robust validation of the functional relevance of PDL-1 expression as a mechanism of resistance.

In conclusion this work represent a novel interrogation of the late effects of signaling pathway inhibitors and immune pathway interaction. This shows small statistical changes in PD-L1 levels, which are not consistent across all cell lines studies.

## Supporting information

S1 Table GI_50_s for cell lines in nM. (DOCX)

S1 File Western blotting of MHC class I in cell lines—Methods and results. (DOCX)

S2 File Validation of PD-1/NFAT reporter- jurkat cell assay. (DOCX)
