# Supplementary material for: A study of PD-L1 expression in KRAS mutant non-small cell lung cancer cell lines exposed to relevant targeted treatments
Source: PLoS One. 2017 Oct 5;12(10):e0186106. doi: 10.1371/journal.pone.0186106 (PMC5628934; doi:10.1371/journal.pone.0186106)
Supplement: S1 Table — (DOCX) [file pone.0186106.s001.docx]

S1 Table GI_50_s for cell lines in nM

|  | AZD5363 | Trametinib |
| --- | --- | --- |
| H441 | 810 | 334.73 |
| H2291 | 1554.9 | 6.76 |
| H23 | 3247.00 | 41.03 |
| H2030 | 3763.33 | 78.61 |
| A549 | 1764.67 | 52.39 |
