# Supplementary material for: A study of PD-L1 expression in KRAS mutant non-small cell lung cancer cell lines exposed to relevant targeted treatments
Source: PLoS One. 2017 Oct 5;12(10):e0186106. doi: 10.1371/journal.pone.0186106 (PMC5628934; doi:10.1371/journal.pone.0186106)
Supplement: S1 File — (DOCX) [file pone.0186106.s002.docx]

Western Blotting of MHC Class I in Cell Lines - Methods

Western blotting was performed to quantify MHC class I expression on the cell lines, to determine if the PD-1/NFAT reporter- jurkat cell line co-culture protocol would be feasible in this context. Lysate of cell lines were prepared and protein concentrations determined using BCA assay. 50μg protein was added to individual wells of the electrophoresis gel and a protein standard was used to visualize molecular weights from 4 – 250 kDa (SeeBlue Plus 2, LC5925; ThermoFisher Scientific; Waltham, MA). After electrophoresis separation (160V for two hours in a Invitrogen Novex Mini Cell Blot Module [ThermoFisher Scientific, Waltham, MA]) and transfer the gel was exposed to antibodies. Primary antibodies used were anti-MHC class I (ab110645, abcam, Cambridge, UK) (1:1000) and GAPDH primary antibody (Anti-GAPDH, MAB 374, Merck Millipore, Billerica, MA) (1:2000). Secondary HRP-conjugate antibodies used were goat anti-rabbit (IR Dye 680RD, Li-COR, Lincoln, NE) (1:3333) and goat anti-mouse (IR Dye 680RD, Li-COR, Lincoln, NE) (1:3333). The electrophoresis membrane was read using a Li-Cor Odyssey FC Imaging system (Li-COR, Lincoln, NE) and exported using Image Studio v5.2 software (Li-COR, Lincoln, NE).

Western Blotting of MHC Class I in Cell Lines - Results

All cell lines demonstrated MHC class I positivity as detailed Figure 1. A549 demonstrated lower levels of MHC class I expression than the other four cell lines.

Figure 1. Western Blot of adeno-NSCLC cell lines with MHC Class I antibody demonstrated at 40kDa and GAPDH at 37kDa
