# Supplementary material for: A study of PD-L1 expression in KRAS mutant non-small cell lung cancer cell lines exposed to relevant targeted treatments
Source: PLoS One. 2017 Oct 5;12(10):e0186106. doi: 10.1371/journal.pone.0186106 (PMC5628934; doi:10.1371/journal.pone.0186106)
Supplement: S2 File — (DOCX) [file pone.0186106.s003.docx]

Validation of PD-1/NFAT reporter- jurkat cell Assay

A cell count dependent decease in luminescence was seen in cell lines on co-culture with the jurkat reporter (Figure 1) A statistically significant increase in luminescence levels were seen in the PD-1/NFAT reporter- jurkat cell line co-culture assay with the addition of PD-L1 neutralizing antibody (#71213; BPS Biosciences, San Diego, CA) (figure 3).

Figure 1. H2030 co-cultured with jurkat reporter cell line (jurkat cell count of 20,000/well)

Figure 2. Luminescence in H2030 co-cultured with PD-1/NFAT reporter- jurkat cell line and PD-L1 neutralising antibody. Comparison by Mann-Whitney.
